# Supplementary material for: Improving Milk Yield, Milk Quality, and Follicular Functionality Behavior in Dairy Cows from the Implementation of Microencapsulated Chili Pepper Supplements in Their Diets
Source: Animals (Basel). 2024 Aug 15;14(16):2361. doi: 10.3390/ani14162361 (PMC11350710; doi:10.3390/ani14162361)
Supplement: Supplementary file 1 [file animals-14-02361-s001.zip › animals-3133016-supplementary.pdf]

Suplementaty materials

Highlights

• Micro-encapsulated hot chili was offered to cross-breed dairy cows

• The consumption of microencapsulated hot chili increases the amount of total solids in milk in crossbred cows.

• The economic return in groups of cows supplemented with microencapsulated hot chili is greater than in cows without this supplementation

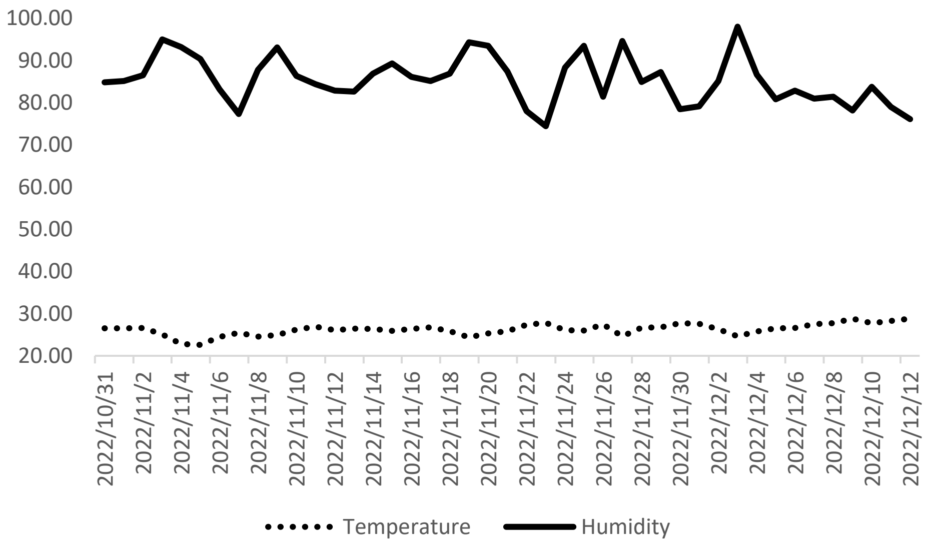

Figure S1. Temperature (°C) and ambient humidity (%) during the experimental period of 42 days.

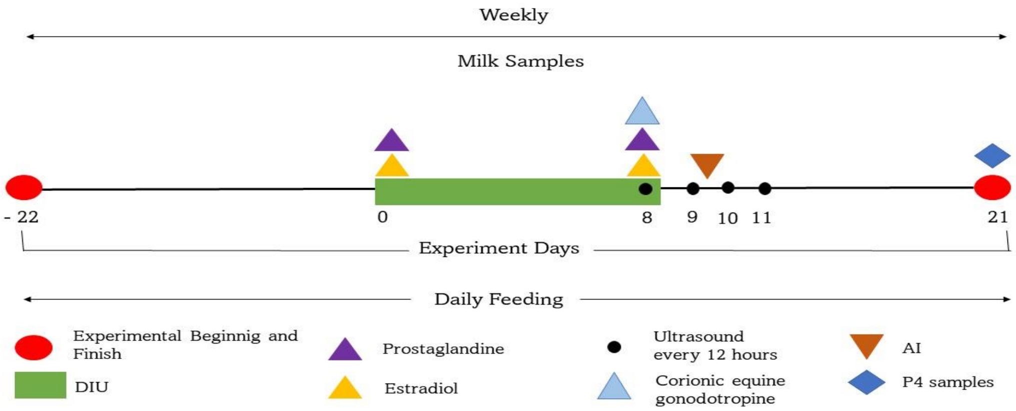

Figure S2. Diagram hormonal protocol. DIU: Progesterone intrauterine device; AI: artificial insemination; P4: Plasma progestere.

Table S1. Milk yield and composition for week in different experimental groups.

Item	Treatment[1]	Week[2]						
		1	2	3	4	5	6	7
	CP	9.10	9.64	9.76	10.43	9.16	8.78	10.27
Milk production (kg/d)	CT	8.20	9.06	9.26	9.43	8.32	7.21	6.57
	P<[3]	0.384	0.573	0.627	0.332	0.416	0.131	0.001
	CT	8.90	9.71	8.16	10.75	8.08	6.14	7.27
FCM, (3.5 %)	CP	8.21	8.46	8.15	10.01	9.72	7.91	9.93
	P<[3]	0.900	0.398	0.996	0.583	0.237	0.245	0.089
	CT	0.03	0.04	0.03	0.04	0.03	0.02	0.02
Fat	CP	0.03	0.03	0.02	0.03	0.04	0.03	0.03
	P<[3]	0.97	0.226	0.886	0.298	0.236	0.395	0.215
	CT	0.02	0.03	0.03	0.03	0.03	0.02	0.02
Protein	CP	0.03	0.03	0.03	0.03	0.03	0.03	0.03
	P<[3]	0.692	0.943	0.733	0.233	0.504	0.274	0.002
	CT	0.04	0.04	0.04	0.04	0.04	0.03	0.03
Lactose	CP	0.04	0.04	0.05	0.05	0.04	0.04	0.04
	P<[3]	0.2709	0.625	0.538	0.233	0.517	0.193	0.002
	CT	0.10	0.11	0.10	0.10	0.10	0.08	0.08
TDE	CP	0.10	0.10	0.11	0.12	0.11	0.10	0.12
	P<[3]	0.735	0.612	0.636	0.139	0.416	0.161	0.001
	CT	0.07	0.08	0.08	0.08	0.07	0.06	0.06
NFDE	CP	0.08	0.08	0.08	0.09	0.08	0.08	0.09
	P<[3]	0.554	0.783	0.614	0.131	0.493	0.213	0.002

	CT	3.39	3.53	3.04	4.03	3.38	2.59	3.19
Fat (%)	CP	3.03	2.69	2.65	3.31	3.90	3.02	3.42
	P<[3]	0.542	0.149	0.545	0.185	0.344	0.481	0.67
	CT	3.06	3.11	3.12	2.97	3.06	3.22	3.24
Protein (%)	CP	2.91	2.94	3.07	3.00	3.00	3.08	3.10
	P<[3]	0.255	0.189	0.669	0.804	0.654	0.304	0.279
	CT	4.50	4.24	4.56	4.57	4.62	4.53	4.55
Lactose (%)	CP	5.43	4.27	4.63	4.70	4.52	4.49	4.48
	P<[3]	0.651	0.884	0.653	0.425	0.499	0.8	0.678
	CT	11.71	11.69	11.22	11.16	12.08	11.27	11.54
TDE (%)	CP	10.92	10.61	11.09	11.84	12014.00	11.48	11.76
	P<[3]	0.118	0.035	0.806	0.195	0.908	0.703	0.675
	CT	8.66	8.49	8.68	8.26	8.67	8.82	8.82
NFDE (%)	CP	8.39	8.28	8.68	8.69	8.52	8.61	8.62
	P<[3]	0.309	0.4074	0.995	0.115	0.566	0.471	0.465
	CT	10.57	12.48	9.98	11.36	8.03	7.49	7.56
MUN (mg/dL)	CP	11.17	10.98	10.67	9.45	8.36	7.74	8.18
	P<[3]	0.594	0.186	0.548	0.106	0.777	0.541	0.593
	CT	153.70	156.98	111.31	129.46	81.23	101.47	53.47
SCC (unit/mL)	CP	192.58	119.23	151.83	143.15	106.74	174.88	196.05
	P<[3]	0.544	0.555	0.498	0.819	0.696	0.278	0.037

Treatment[1]= CT= Control (no chili pepper micro encapsuled); CP= pepper micro encapsuled supplemented.

Week[2]= Week of experiment.

[3]Probability effect for group. week. and interaction between group and week (Inter).

FCM= Fat corrected milk calculated with using the following equation (Sklan et al.. 1992); TDF=Total dry extract; NFDE=Non-fat dry extract; MUN= Milk urea nitrogen; SCC= Somatic cell count.
